# Optical Fiber FP Sensor for Simultaneous Measurement of Refractive Index and Temperature Based on the Empirical Mode Decomposition Algorithm

**DOI:** 10.3390/s20030664

**Published:** 2020-01-24

**Authors:** Everardo Vargas-Rodriguez, Ana Dinora Guzman-Chavez, Roberto Baeza-Serrato, Mario Alberto Garcia-Ramirez

**Affiliations:** 1 Departamento de Estudios Multidisciplinarios, División de Ingenierías, Campus Irapuato-Salamanca, Universidad de Guanajuato, Av. Universidad s/n, Col. Yacatitas, Yuriria, Guanajuato C.P. 38940, Mexico; r.baeza@ugto.mx; 2 Centro Universitario de Ciencias Exactas e Ingenierías, Universidad de Guadalajara, Blvd. Marcelino Garcia Barragan 1421, Guadalajara, Jalisco C.P. 44430, Mexico; mario.garcia@academicos.udg.mx

**Keywords:** empirical mode decomposition, refractive index, temperature, sensors, optical fiber

## Abstract

In this work, a dual refractive index and temperature sensor based on an interferometric system and on the empirical mode decomposition (EMD) algorithm is presented. Here, it is shown that the EMD provides a comprehensive way to analyze and decompose complex reflection spectra produced by an interferometric filter build at the tip of an optical fiber. By applying the EMD algorithm, the spectrum can be decomposed into a set of intrinsic mode functions (IMF) from which the temperature and the refractive index can be easily extracted. Moreover, the proposed methodology provides a detailed insight of the behavior of this type of interferometric sensors and allows widening of the dynamic measurement ranges of both variables. Here, for proof of principle purposes, a filter based on a stack of three layers (two of them were thermo-sensitive) was fabricated. Finally, it is shown that the proposed methodology can decompose the experimental measured spectra and to determine the refractive index and the temperature, supporting the mathematical model.

## 1. Introduction

In recent decades, several optical sensor designs for measuring refractive index have been proposed. For instance, Urrutia et al. [1] recently presented a comprehensive review of optical fiber refractrometers in which a considerable number of different setups are analyzed. Some interesting sensor designs are those based on optical fibers in which the sensing element is fabricated at the tip of the fiber. From these, the sensors based on the Fabry-Perot Interferometer (FPI) are particularly attractive since these can be easily fabricated. Typically, the FPI can be implemented by forming an air cavity at the tip of the optical fiber which can be sealed [2,3,4,5,6] or open to the external medium [7,8]. The reflection spectrum of these FPIs is formed by a set of fringes which can be described by a quasi-sinusoidal waveform. Here, the amplitude of these fringes will depend directly on the indexes of refraction of both the materials forming the FPI’s cavity and the exit medium surrounding the FPI. Commonly, when the FPI’s cavity is sealed and its mirrors are of silica, only the amplitude of the fringes depends directly on the refractive index of the exit medium while the free spectral range (FSR) only depends on the thickness of the cavity. In contrast, when the FPI’s cavity is open, the amplitude, the position of fringes, and the FSR of the fringes will vary depending on the refractive index of the exit medium. Other FPIs designs are based on a set of films deposited at the tip of the optical fiber [9,10,11]. Here, the complexity of the overall spectrum of the reflected intensity distribution of the interferometer increases with the number of films, since it is formed by the superposition of several spectra which are due to multiple reflections occurring between the interfaces of the layers. Moreover, in these designs different materials can be deposited allowing us to control the visibility of the spectral fringes. Furthermore, the layers materials can be selected to be thermo-sensitive to induce effects in the spectral fringe pattern due to temperature. This can be useful to determine simultaneously different variables, such as the refractive index and temperature.

For dual sensing applications the complexity to model and to determine the values of each variable from a single spectrum can be increased if this is formed by the superposition of different spectra, which change their characteristics due to the temperature, the relative humidity [10] or the refractive index of the exit medium. For these cases, it is quite important to systematically relate the changes observed in the reflected spectrum with the physical variables to be measured. To perform the measurements, some authors have proposed to establish linear relationships between the physical variables and either the dip position of some fringes [12] or the intensity or the shift of a resonant dip wavelength [5]. Other authors have proposed to use the Fourier transform to determine the harmonic components of a FPI’s reflection spectrum and relate them with a physical variable [10]. This type of approximation has proved to be simple and that the refractive index and the temperature can be estimated with high sensitivity, but usually with a very limited dynamic measurement range. For instance, in [13], a dual sensor was implemented by depositing a layer of poly-dimethylsiloxane (PDMS). The refractive index and temperature measurements were performed by tracing the peak and the position of a spectral fringe. In that way, authors achieved dynamic operating ranges of 1.3625–1.4150 RIU and 25–60 °C. Similarly, in [14], authors presented a sensor which was fabricated by depositing a layer of UV-curable polymer at the tip of an optical fiber and for performing the refractive index measurement, they evaluated the contrast of the spectral fringes and for temperature measurement, the shift of a spectral dip was traced. Following that procedure, authors achieved dynamic ranges of 1.38–1.42 and 24–55 °C. Additionally, they showed that the contrast of their spectral fringes was minimum when the refractive index of the exit medium was around 1.45. Another example of a dual sensor is the proposed in [12] which was implemented by splicing an isopropanol infiltrated hollow core fiber between two single-mode fibers. In that work, authors traced a dip of the spectrum to perform the temperature and refractive index measurements for operating dynamic ranges of 25–80 °C and 1.333–1.376, respectively. Hence, for simultaneous measurement applications it is compulsory to model the behavior of the spectral pattern to validate the dynamic ranges of temperature and refractive index that can be measured by using linear relationships. This is important because of the spectral fringes observed in the reflected intensity distribution spectrum can have different behavior depending on the combination of the refractive index and temperature values. Consequently, the relationship between the fringe intensity and its position with the temperature and the refractive index can be non-lineal for relatively wide dynamic ranges.

In this work, a new methodology for refractive index and temperature sensing, based on the Empirical Mode Decomposition (EMD) algorithm, is presented. This methodology can be of interest for interferometric sensors in which the overall reflected intensity distribution pattern is formed by the superposition of several spectra. Moreover, the methodology consists on the decomposition of a complex spectrum into a set of intrinsic mode functions (IMFs) and allowed us to establish comprehensive relationships between some characteristics of these functions and the temperature and the refractive index. Hence, these IMFs provide us a detailed insight about the behavior of each superimposed spectra and consequently allowed us to determine the refractive index and the temperature over wide dynamic ranges. Finally, a filter with two thermal-sensitive layers was fabricated and a sensor was implemented, later it is shown that the experimental results agree with the theoretical calculations supporting the proposed methodology.

## 2. Sensing Element Design and Fabrication

**Figure 1:**
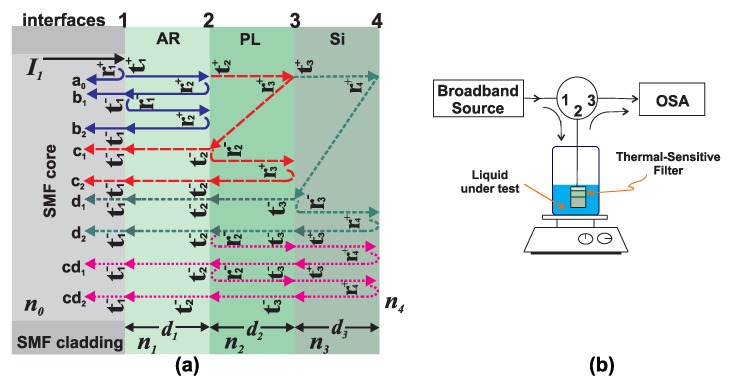
(**a**) Sketch of the reflected and transmitted waves occurring at each interface of the tunable FP filter; (**b**) sensing optical setup.

The interferometric sensor head fabricated consisted on a stack of three layers at the tip of an optical fiber (Figure 1a). Two of these layers are thermo-sensitive, one of them is of a cyanoacrylate polymer (PL) while the other one is of silicon (Si). The third layer is an antireflection (AR) coating at the tip of a single-mode optical fiber. In this form basically three optical cavities are formed and therefore the overall reflected intensity distribution pattern of the filter will be formed by the superposition of different spectra due to the multiple internal reflections occurring between the interfaces of the cavities. Moreover, the overall reflected spectrum of the filter will depend on both the temperature and the refractive index of the exit medium $\left(n_{4}\right)$ since it forms an optical interface with the silicon layer (Figure 1a).

The reflected intensity distribution pattern of the filter can be mathematically modeled as the superposition of waves a${}_{1}$, $b_{k}$, $c_{k}$ and $d_{k}$, which are the *k*-th reflection occurring at interfaces *m* = 1,2,3,4, respectively (Figure 1a). Moreover, the contribution of multiple reflections occurring between two not consecutive neighbor interfaces are described by $c_{dk}$. Hence, the amplitude of the electric vector of the reflected light can be expressed as:

(1) $$\begin{array}{ccc} I_{r} & = & I_{i}[r_{1}^{+}+t_{1}\sum_{k=1}^{N}{\left[L_{1}r_{2}^{+}\right]}^{k}{\left(r_{1}^{-}\right)}^{k-1}e^{ik\delta_{1}}+t_{1}t_{2}L_{1}\sum_{k=1}^{N}{\left[L_{2}r_{3}^{+}\right]}^{k}{\left(r_{2}^{-}\right)}^{k-1}e^{i(\delta_{1}+k\delta_{2})}\\ & + & t_{1}t_{2}t_{3}L_{1}L_{2}\sum_{k=1}^{N}{\left[L_{3}r_{4}^{+}\right]}^{k}{\left(r_{3}^{-}\right)}^{k-1}e^{i(\delta_{1}+\delta_{2}+k\delta_{3})}+t_{1}t_{2}L_{1}\sum_{k=1}^{N}{\left[L_{2}L_{3}t_{3}r_{4}^{+}\right]}^{k+1}{\left(r_{2}^{-}\right)}^{k}e^{i[\delta_{1}+\left(k+1\right)\left(\delta_{2}+\delta_{3}\right)]}\\ & + & t_{1}\sum_{k=1}^{N}{\left[L_{1}L_{2}L_{3}t_{2}t_{3}r_{4}^{+}\right]}^{k+1}{\left(r_{1}^{-}\right)}^{k}e^{i\left(k+1\right)\left(\delta_{1}+\delta_{2}+\delta_{3}\right)}+t_{1}\sum_{k=1}^{N}{\left[L_{1}L_{2}t_{2}r_{3}^{+}\right]}^{k+1}{\left(r_{1}^{-}\right)}^{k}e^{i\left(k+1\right)\left(\delta_{1}+\delta_{2}\right)}]. \end{array}$$

The parameters used in Equation (1) are defined in Table 1. Afterwards, the overall intensity distribution of the reflected spectrum of the filter can be calculated as $R=|I_{r}/I_{i}{|}^{2}$, where $I_{i}$ is the incident wave at interface 1 (Figure 1a). Finally, the optical sensing arrangement used in this work is presented in Figure 1b.

### 2.1. Characterization of Reflected Intensity Distribution Pattern of the Fabricated Filter

**Figure 2:**
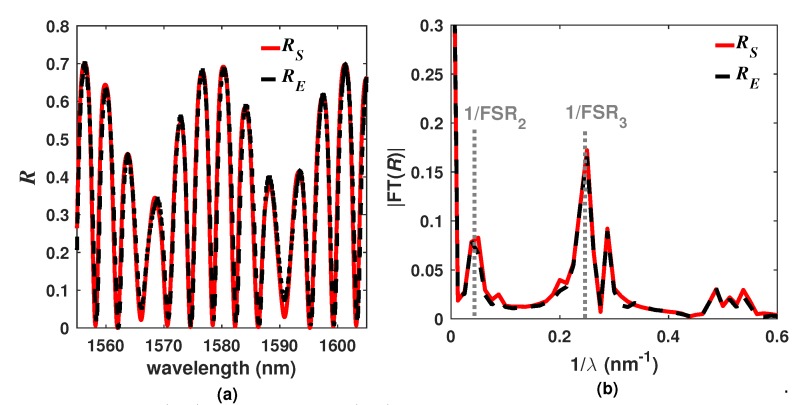
(**a**) Simulated $\left(R_{S}\right)$ and measured $\left(R_{E}\right)$ reflected intensity distributions patterns of the filter, considering a $n_{4}=1$ and T=25°C, and (**b**) magnitude of their Fourier transforms.

The optical filter was implemented by using an optical fiber ferrule with an antireflection coating which forms the first layer of the filter (p=1). The second layer (p=2) was of cyanoacrylate polymer and finally the third layer (p=3) was formed with a silicon wafer (Figure 1a). The fabrication process was previously described in detail by authors in [11]. Moreover, the materials properties of each layer and their experimental thicknesses are listed in Table 2. Once the filter was fabricated, it was characterized by using the optical setup shown in Figure 1b, in which the light from a broad band source enters into the port 1 of an optical circulator and travels to the port 2 in such a way that it arrives to the optical filter and its reflection gets back by the port 2 and leaves through the port 3 where it is recorded with an optical spectrum analyzer (OSA). The experimental optical losses were estimated by using the cost function $CF={\left(\sum_{j=0}^{M}{\left[R_{E}\left(\lambda \right)-R\left(\lambda ,A_{1},A_{2},A_{3}\right)\right]}^{2}\right)}^{1/2}$ [15], where 0$\leq A_{p}\leq$1 and *M* is the number of wavelength samples of the experimental spectrum ($R_{E}$). For instance, the simulated and measured reflected intensity distribution spectra of the filter F1 are shown in Figure 2a, here the exit refractive index was fixed to $n_{4}=1$ and the temperature at $T=25^{\circ }$C. It can be appreciated that the simulated spectrum ($R_{S}$) agrees with $R_{E}$. Furthermore, it can be clearly observed that the overall spectrum *R* is formed by the superposition of two main spectra. One of these is due to the PL layer and therefore it has a free spectral range FSR2≈22.05 nm and the second one is due to the Si layer with a FSR3≈4.16 nm. This can be confirmed by calculating the Fourier transform of the spectra *R*, in which two main sidelobes centered at 1/FSR_2_ and 1/FSR_3_ (Figure 2b) can be appreciated.

### 2.2. Characterization of the Refractive Index Effects

**Figure 3:**
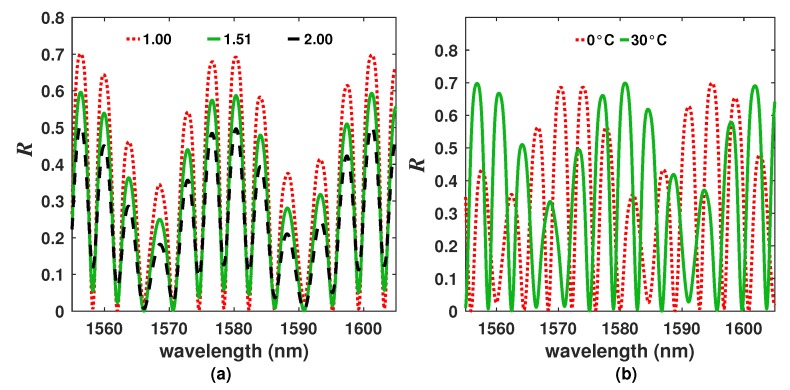
(**a**) Simulated intensity distribution of the reflected spectrum ($R_{S}$) considering different $n_{4}$ values and T=25°C, and (**b**) simulated spectrum for $n_{4}=1$ and different *T* values.

To characterize the effects of the refractive index of the exit medium over the overall *R* spectrum, the refractive index of the exit medium was varied 1$\leq n_{4}\leq$2, while the temperature was kept fixed. For this case, it was observed that the peak to peak amplitude of the fringes decreases (Figure 3a) as the exit refractive index increases. Indeed, this tendency will be maintained for the refractive index range within 1$\leq n_{4}\leq$3.4 which is an advantage since there is not a change of tendency around 1.45.

### 2.3. Characterization of the Temperature Effects

Now, as a first approach, the temperature effects over the overall *R* spectrum were simulated considering that the exit refractive medium was fixed at $n_{4}=1$ while the temperature is varied. Here, the spectrum is shifted to the right as the temperature increases (Figure 3b). Moreover, it was observed that when the temperature changed from 0 to ≈80${}^{\circ }$C, the spectrum was shifted approximately 22.05 nm (≈1 FSR_2_). Furthermore, the spectral fringe distribution changes because both superimposed spectra (due to Si and PL layers) shift at different rates. This is because both layers have different thermo-expansion and thermo-optic coefficients as well as different thicknesses, inducing a complex behavior of the spectrum.

### 2.4. Simultaneous Refractive Index and Temperature Effects

**Figure 4:**
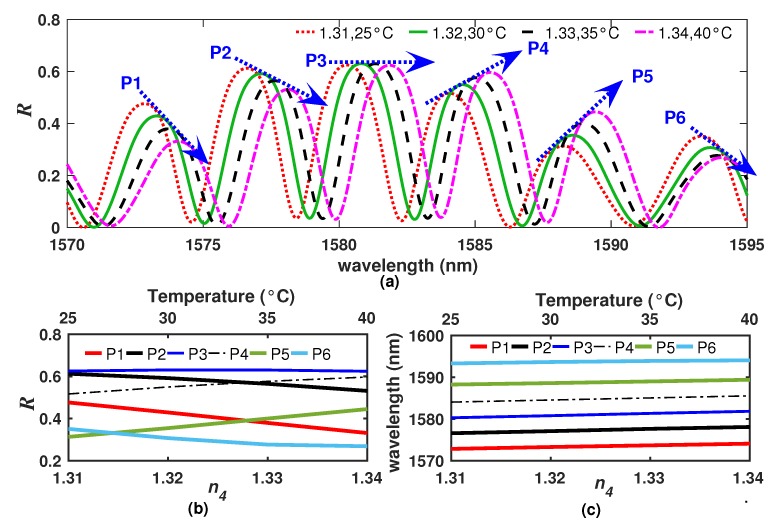
(**a**) Detail of the simulated *R* spectrum considering different $n_{4}$ and *T* values; (**b**) peak amplitude and (**c**) peak positions of the fringes P1 to P6 as $n_{4}$ and *T* are varied.

As the *R* spectrum is affected by $n_{4}$ and *T*, it was performed a simulation considering that both variables change simultaneously. This can be of interest for different applications for which the refractive index of the material changes with the temperature [20,21,22]. Ideally, for a filter with a intensity distributions of the reflected patterns spectrum with only a main FSR, the amplitude of the fringes must depend only on the refractive index $n_{4}$ and the wavelength shifting on the temperature. However, as the *R* spectrum of our filter is composed of two main spectra with different FSRs and spectral shifting rates, both the peak amplitude and the peak position will depend on the combination of $n_{4}$ and *T*. Therefore, each fringe can have a different sensitivity for these combinations, in such a way that the peak amplitude of some fringes can either increase, decrease or remain almost constant for the same combinations of $n_{4}$ and *T*. For instance, in Figure 4a, a detail is shown of the *R* spectra considering different $n_{4}$ and *T* values. In this figure, it can be observed 6 fringes that we labeled as P1 to P6. Of these, the peak amplitude of fringes P1, P2, and P6 decreases as $n_{4}$ and *T* increases. By the contrast, for the same conditions of $n_{4}$ and *T*, the peak amplitude of the fringes P4 and P5 increases while the peak of the P3 remains almost fixed. In these simulations, values of $n_{4}$ between 1.31 and 1.34 and of *T* between 25 and 40 ${}^{\circ }$C were considered. Moreover, in Figure 4b, it can be observed that the rate of change of the amplitude of the fringes have different slopes (with positive and negative tendencies) and therefore not all the fringes have the same sensitivity to $n_{4}$ and *T* within the same dynamic ranges. Now, peak positions are shifted to the right as the temperature increases and all presented practically the same temperature sensitivity (Figure 4c). This behavior can be explained by the fact that the two thermo-sensitive layers and the exit refractive index contribute in the overall *R* spectrum.

**Figure 5:**
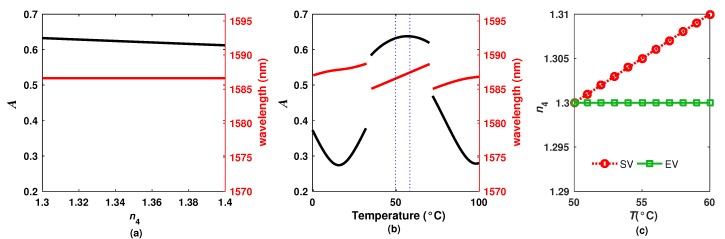
Simulated peak amplitude of the fringes (**a**) P1 and (**b**) P3 as a function of $n_{4}$ and *T*; (**c**) Reference set of refractive index and temperature values (SV) considered in the simulations compared with the estimated values (EV) obtained with the dual-parameter sensing matrix.

For sensing applications, the fact that each fringe has different sensitivities to $n_{4}$ and *T* can represent a problem since it is necessary to define the dynamic ranges for which the sensitivity is valid. Moreover, in some cases it is not possible to establish linear relationships between the position and the amplitude of one fringe with the temperature and the refractive index. For example, let us to consider the behavior of the fringe P4 (Figure 4a) over wider dynamic ranges. First, it is considered that the temperature is fixed (*T* = 50 °C) while the refractive index is varied within 1.3 $\leq n_{4}\leq$1.4 to trace the fringe peak amplitude and its wavelength position amplitude (Figure 5a). Here, it can be appreciated that the amplitude of the peak decreases as $n_{4}$ increases, while the wavelength position remains constant. Secondly, the results obtained when we consider that the refractive index was fixed to $n_{4}$ = 1.3 while the temperature was varied within 0 ≤T≤ 100 °C are presented in (Figure 5b). Here, it can be appreciated that both parameters, the fringe wavelength position and its amplitude, are affected simultaneously by temperature. Additionally, this effect presents a non-linear behavior. In this case, if we would like to establish linear relationships for the dynamic ranges of $1.3\leq n_{4}\leq 1.31$ and 50≤T≤60 °C, it is possible to determine from Figure 5a that the refractive index sensitivities to the amplitude and to the wavelength position of the fringe are $\kappa_{A,n}∼-2.0375\times 10^{-1}$/RIU and $\kappa_{\lambda ,n}∼$1.725$\times 10^{-2}$ nm/RIU, respectively. Moreover, for these dynamic ranges, the temperature sensitivities to the amplitude and to the wavelength of the fringe are $\kappa_{A,T}∼$4.114$\times 10^{-3}$/°C and $\kappa_{\lambda ,T}∼$1.0398 nm/°C, respectively (Figure 5b). Afterwards, the refractive index and the temperature can be estimated by applying the dual-parameter sensing matrix [13]:

(2) $$\left[\begin{array}{c} \Delta n\\ \Delta T \end{array}\right]={\left[\begin{array}{cc} \kappa_{A,n} & \kappa_{\lambda ,n}\\ \kappa_{A,T} & \kappa_{\lambda ,T} \end{array}\right]}^{-1}\left[\begin{array}{c} \Delta A\\ \Delta \lambda \end{array}\right]$$

To test this approximation, a set of combinations values of $n_{4}$ and *T* was defined and we labeled as SV (Figure 5c). The intensity distribution of the reflected spectrum (*R*) for each combination of the SV set was calculated and the amplitude (*A*) and the position (λ) of the peak was recorded. Afterwards, these values were introduced into Equation (2) to obtain a set of estimated values of $n_{4}$ and *T*, we labeled this set as estimated values (EV). The comparison between the SV and the EV results are presented in Figure 5c, in which it can be observed that the temperature is estimated correctly however the refractive index estimation rapidly diverges, which is due to the strong cross sensitivity of $n_{4}$ and *T* since two of the layers of the filter are sensitive to temperature. Therefore, the method of tracing the amplitude and the wavelength of the fringe to determine simultaneously the temperature and the refractive index over relatively wide dynamic ranges is not the best option for our sensor. Hence, the implementation of a methodology based on the Empirical Mode Decomposition (EMD) algorithm is proposed. This algorithm allowed us to decompose the overall *R* spectrum of the filter into a set of functions, which provide us a detailed insight information about the behavior of each component of the spectrum permitting us to enhance the simultaneous sensing of different variables, as in our case *n* and *T*.

## 3. Spectrum Decomposition

**Figure 6:**
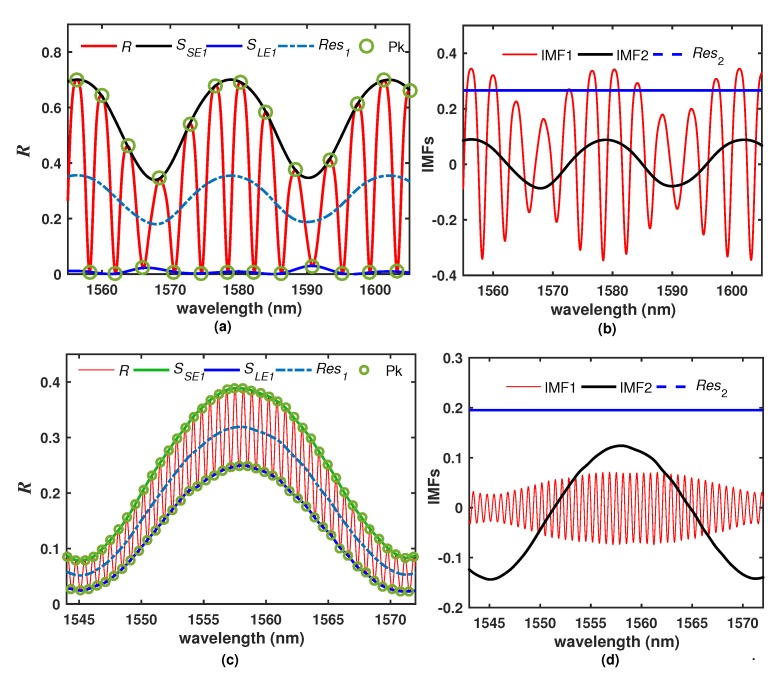
(**a**) Simulated (R) pattern of the filter F1 and the calculated signals required for decomposing this spectrum by using the EMD algorithm and (**b**) obtained IMFs and residual function; (**c**) Example of the experimental *R* pattern of the filter F2 and its corresponding (**d**) IMFs and residual function.

There are different techniques to know the harmonic composition of a periodic signal, for example, a broadly used method is the Fourier Transform. In our case, we will use the Empirical Mode Decomposition (EMD) which is a widely used technique for signal analysis processing since it is a powerful tool to decompose an original signal into a set of AM/FM zero-mean components called Intrinsic Mode Functions (IMF). Additionally, a remainder function with non-zero mean will be obtained [23,24]. The EMD algorithm is explained with detail in elsewhere [23,24,25], but for the sake of the discussion of our application, we will just to list the most important steps of the EMD procedure: 1.- A counter is initialized as j=1 and the overall *R* spectrum is defined as the input signal $S_{j}=R$; 2.- The peaks and valleys of $S_{j}$ are localized; 3.- One vector with the peaks and another one with the valleys points are formed. 4.- A cubic spline interpolation of the peaks points (Pk) of each one of the vectors is performed in order to obtain the superior envelope $\left(S_{SEj}\right)$ and the lower envelope $\left(S_{LEj}\right)$ (Figure 6a); 5.- The mean of the envelopes $\left(Res_{j}\right)$ is calculated (Figure 6a); 7.- The function $F_{j}=S_{j}-Res_{j}$ is calculated and if $F_{j}$ is a zero-mean function, the step 8 is executed, otherwise it jumps to step 9; 8.- The function $F_{j}$ is accepted as an IMF, therefore set IMF$j=F_{j}$ (Figure 6b), j=j+1, as the new input signal set $S_{j}=Res_{j-1}$ and repeat the procedure from step 2 until $Res_{j}$ get as a function which is either constant, monotonic or it has only one maximum and a minimum, from which no an IMF can be extracted (Figure 6b); 9.- Set $S_{j}=F_{j}$ and repeat the procedure from step 2. Following this procedure, the *R* spectrum can be expressed in terms of its IMFs as:

(3) $$R=\sum_{j=1}\mathrm{IMF}j+Res_{j}$$

As an example, the overall *R* spectrum of filer F1 (Table 2) was decomposed into two intrinsic mode functions (IMF1 and IMF2) and one residual function obtained from $Res_{2}$ (Figure 6b). The IMF1 has the narrowest FSR since it is related to the Si layer, while IMF2 is related to the PL layer and consequently it has a much wider FSR. As a further example, the measured reflected intensity pattern of the filter F2 (Table 2), which it is shown in Figure 6c, was also decomposed by applying the EMD algorithm and the results are presented in Figure 6d. In this filter the IMF1 has a shorter FSR compared with the obtained for the filter F1; this is due to the F2 has a layer 3 considerably thicker than the used in filter F1. Finally, it is important to mention that one advantage of the EMD algorithm is its capability to process noisy signals. In that case, the noise is separated from the spectrum and returned as an additional high frequency IMF [23,24,25].

## 4. Effects of the Refractive Index and the Temperature over the IMFs

**Figure 7:**
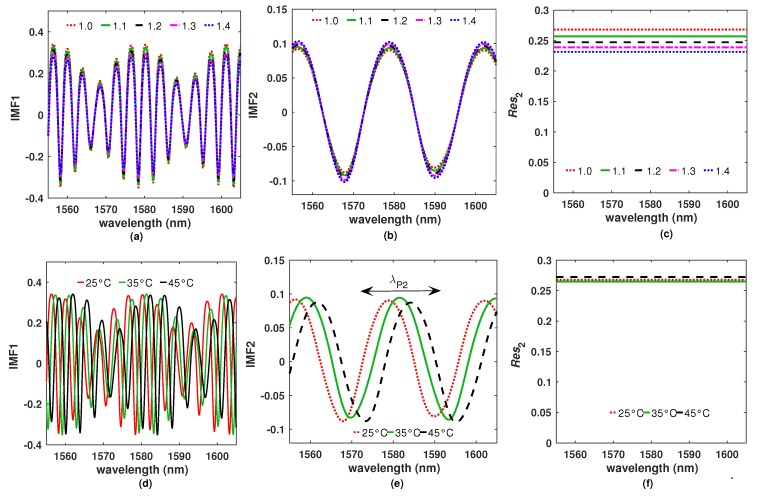
*R* spectra decomposed into (**a**) IMF1, (**b**) IMF2 and (**c**) $Res_{2}$ for different $n_{4}$ values and T=25 °C. Simulated (**d**) IMF1, (**e**) IMF2 and (**f**) $Res_{j}$ considering n=1 and different *T* values.

To understand the effect of $n_{4}$ and *T* over the IMFs, some *R* spectra of the filter F1 were calculated and decomposed by using the EMD algorithm. The IMFs (IMF1, IMF2) and the residual functions $Res_{2}$ were calculated considering a fixed value of T=25 °C while $n_{4}$ was varied (Figure 7a–c). Moreover, a further *R* spectra set was simulated setting $n_{4}=1$ while *T* was varied and their corresponding IMFs functions are presented in Figure 7d–e. Here, it can be observed that the amplitude of the IMF1 function varies rapidly as $n_{4}$ changes (Figure 7a). Moreover, this function also shifts rapidly with temperature (Figure 7d). In contrast, the amplitude of the IMF2 function do not change considerably with $n_{4}$ (Figure 7b) and just shifts to the right as the temperature increases (Figure 7e). This can be explained by the fact that the IMF2 function is due to multiple reflections occurring within the PL layer, and therefore the amplitude of the fringes will depend directly on the refractive indexes of its optical interfaces which are not affected by the refractive index of the exit medium. Furthermore, the change in the PL layer thickness is the main effect produced by the temperature variation which consequently induces the spectral shifting of the IMF2 function.

**Figure 8:**
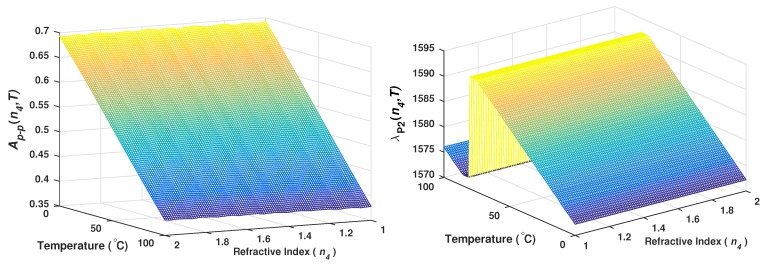
(**a**) Peak to peak amplitude of the IMF1 and (**b**) peak position of the IMF2 as a function of $n_{4}$ and *T*.

Based on the knowledge of the IMFs behavior as a function of the temperature and the refractive index, it is possible to establish different relationships between the variables and some characteristics of these functions. As an example, the relationship between the peak to peak amplitude $\left(A_{p-p}\right)$ of the IMF1 function and the combination of $n_{4}$ and *T* was evaluated. Additionally, from the IMF2 the wavelength position of the fringe’s peak ($\lambda_{P2}$) as a function of *T* and $n_{4}$ was determined. Here, the peak position of the IMF2 was traced within one spectral window limited by one FSR_2_; in our case, this window was limited within the wavelength range from 1572 to 1593 nm. The results of $A_{p-p}\left(n_{4},T\right)$ and of $\lambda_{P2}\left(n_{4},T\right)$ are shown in Figure 8a,b, respectively.

**Figure 9:**
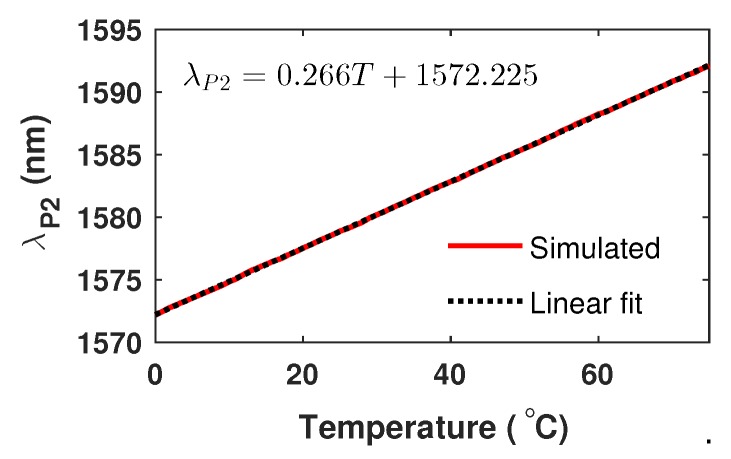
Wavelength position of $\lambda_{P2}$ as a function of *T*.

From these results, it is important to observe that the peak of the IMF2 function ($\lambda_{P2}$) shifts lineally with the temperature and it is quasi-insensitive to $n_{4}$. Moreover, $\lambda_{P2}$ returns to its initial position when the temperature reached a change of ΔT≈76°C (Figure 8b). Here, the behavior of $\lambda_{P2}\left(n_{4},T\right)$ for the temperature range 0≤T≤76 °C and for any value of $n_{4}$ is shown in Figure 9. In this figure, it can be observed that $\lambda_{P2}$ shifts lineally with the temperature and their relationship can be expressed as:

(4) $$T=3.759(\lambda_{P2}-1572.225)$$

Now, as the $A_{p-p}$ and *T* experimental values can be calculated directly from the IMFs of the reflection spectrum, therefore, it is possible to perform a polynomial surface fit to establish the relationship of $n_{4}$ as a function of $A_{p-p}$ and *T* (Figure 8a). Moreover, the precision achieved can be increased depending on the degree of the polynomial fit. For demonstration purposes a polynomial fit of degree 1 was considered to calculate the experimental refractive index of the exit medium, and for our sensor it was obtained that:

(5) $$n_{4}=8.3563\times 10^{-5}T-3.1651A_{p-p}+3.1939.$$

The relationships expressed by Equations (4) and (5) means that if the measurement are obtained by using this process the sensor will have a linear response when it operates within the dynamic ranges of 1$\leq n_{4}\leq$2 and 0≤T≤76 °C. Moreover, it is clear that following this methodology it is possible to establish a comprehensive mathematical model of the sensor response to determine simultaneously the refractive index of the exit medium and its temperature over wide dynamic ranges from a single *R* spectrum. Furthermore, this methodology gives us a detailed insight of the behavior of each superimposed spectrum and therefore it can be applied to other sensors based on filters with a similar spectral response.

## 5. Experimental Measurement of Refractive Index and Temperature

**Figure 10:**
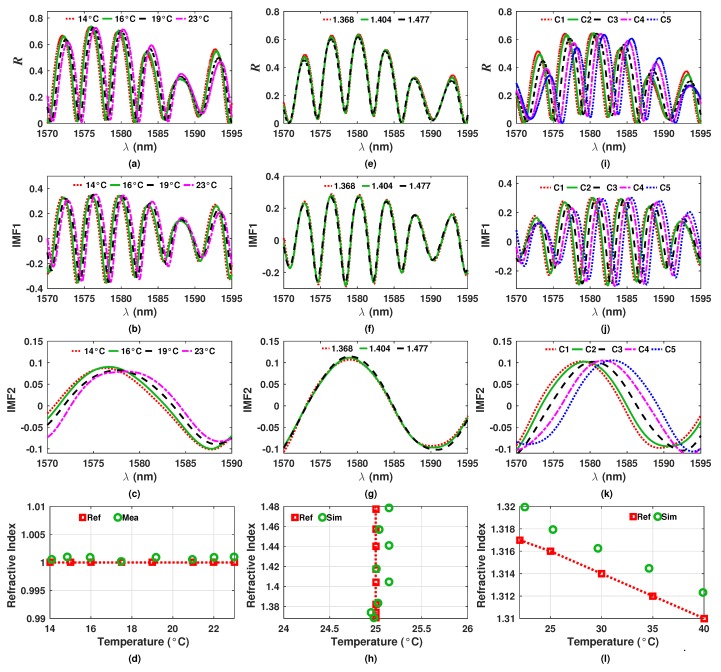
(**a**) Experimental *R* spectrum of the filter in air and at different temperatures and their corresponding (**b**) IMF1 and (**c**) IMF2 functions and (**d**) the estimated $n_{4}$ and *T* values; (**e**) Experimental *R* spectrum at T=25 °C and different $n_{4}$ values and their corresponding (**f**) IMF1 and (**g**) IMF2 functions and (**h**) the estimated $n_{4}$ and *T* values; (**i**) Experimental *R* spectrum at different *T* and $n_{4}$ values and their corresponding (**j**) IMF1 and (**k**) IMF2 functions and (**l**) the estimated $n_{4}$ and *T* values.

To verify the viability of the application of the EMD algorithm for decomposing the *R* spectrum of the interferometric sensor, some experimental measurements were performed. Here, to define a reference group of liquids, some samples were characterized by using a commercial refractometer with temperature controller to determine their experimental refractive index at certain temperature. Afterwards the experimental setup presented in Figure 1b was implemented, where the reference sample was heated by using a hotplate with a resolution of 1 °C. Moreover, a thermocouple with an accuracy of ±1.1 °C was used to monitor the temperature of the liquid sample. The first set of measurements consisted on let the filter in air ($n_{4}=1$) while the temperature was varied. The measured spectra for these conditions are shown in Figure 10a. Afterwards, these spectra were decomposed into their first (IMF1) and second intrinsic (IMF2) mode functions (Figure 10b,c). From these figures, it can be appreciated that the IMFs obtained from experimental measurements are quite similar to the simulated and presented previously. Once the IMFs were obtained, the temperature (*T*) was calculated by determining the wavelength position of the fringe peak of the IMF2 ($\lambda_{P2}$) and by substituting this value into Equation (4). Later, the peak to peak amplitude $\left(A_{p-p}\right)$ of the IMF1 was calculated. Finally, the experimental $n_{4}$ can be calculated by substituting both values, *T* and $A_{p-p}$, into Equation (5). The experimental results and the reference values are shown in Figure 10d. Here, it can be appreciated that the results obtained experimentally by using the proposed methodology are in good agreement with the reference values. 

In the second test, the temperature was fixed at T=25 °C while the exit refractive index was varied between 1.368 and 1.477. Some examples of the measured *R* spectra are shown in Figure 10e while their IMF1 and IMF2 functions are shown in Figure 10f,g, respectively. From these functions the $n_{4}$ and *T* were calculated and the results are presented in Figure 10h. Here, it is important to point out that we consider that the error between the reference temperature and the obtained with our sensor is due to the accuracy of the instruments used in the experiments. Finally, in the third experiment both temperature and refractive index were varied simultaneously. Here, we set the following five combinations of *T* and $n_{4}$: C1: 22 °C and 1.317, C2: 25 °C and 1.316, C3: 30 °C and 1.314, C4: 35 °C and 1.312 and C5: 40 °C and 1.310. The measured *R* spectra for these combinations are exhibited in Figure 10i while their intrinsic functions are shown in Figure 10j,k. After, from these functions the values of *T* and $n_{4}$ were estimated (Figure 10l). This last experiment is important because it allowed us to determine the refractive index and the temperature of the sample which was not possible by directly tracing the wavelength position and the amplitude of a fringe of the spectrum, due to the strong cross sensitivity between the two variables. Considering these experimental measurements it can be concluded that the proposed methodology, based on the EMD algorithm, provides an excellent option to decompose complex spectra formed by the superposition of different spectra. In our case, these spectra are due to the layers of our interferometric filter. Finally, this methodology can be expanded for other interferometric sensors in which the physical variables to be determined have a contribution over the intensity distribution pattern.

## 6. Conclusions

In this work, it was demonstrated that multi-layer filters with more than one thermal-sensitive layer can be useful to measure simultaneously temperature and refractive index. Moreover, it was shown that this type of filter presents a quite complex reflected intensity distribution pattern that depends on both variables. Therefore, for determining simultaneously their values, a novel methodology based on the Empirical Mode Decomposition algorithm was implemented. By using this method, a single *R* spectrum is decomposed into a couple of intrinsic mode functions from which the temperature and the refractive index is determined. Finally, this methodology can be used for other interferometric sensors that have *R* spectra with a complex behavior caused by more of one physical variable.

## Figures and Tables

**Table 1:** List of parameters used in equation 1 for the *m*-th interface and the *p*-th layer (Figure 1).

Description	Parameter
Refractive index	$n_{p}$
Layer thickness	$d_{p}$
Thermo-optic coefficient	$\rho_{p}$
Thermo-expansion coefficient	$\gamma_{p}$
Phase difference	$\delta_{p}=4\pi \left(n_{p}+\Delta n_{p}\right)\left(d_{p}+\Delta d_{p}\right)/\lambda$
Optical losses	$L_{p}=1-A_{p}$
Refractive index change	$\Delta n_{p}=\rho_{p}\left(T-T_{0}\right)$
Layer thickness variation	$\Delta d_{p}=d_{p}\gamma_{p}\left(T-T_{0}\right)$
Transmission coefficients	$t_{m}^{+}=t_{m}^{-}=2n_{m-1}/\left(n_{m-1}+n_{m}\right)$
Reflection coefficients	$r_{m}^{+}=-r_{m}^{-}=\left(n_{m-1}-n_{m}\right)/\left(n_{m-1}+n_{m}\right)$

**Table 2:** Values of the material properties of each layer of the fabricated filters.

Parameter	F1	F2
$\gamma_{1}$	0	
$\gamma_{2}$	198 μm/(m K) [16]	
$\gamma_{3}$	2.6μm/(m K) [17]	
$\rho_{1}$	0	
$\rho_{2}$	$-0.4\times 10^{-4}$ K${}^{-1}$	
$\rho_{3}$	$1.88\times 10^{-4}$ K${}^{-1}$ [18]	
$n_{0}$	1.44	
$n_{2}$	1.45	
$n_{3}$	$3.41696+0.138497{\left(\lambda^{2}-0.028\right)}^{-1}$	
	$+0.013924{\left(\lambda^{2}-0.028\right)}^{-2}-2.09\times 10^{-5}\lambda^{2}$	
	$+1.48\times 10^{-7}\lambda^{4}+1.5\times 10^{-4}\left(T-T_{0}\right)$ [19]	
$n_{1}d_{1}$	387.48 nm	
$A_{1}$	0	0.47
$A_{2}$	0.01	0
$A_{3}$	0.19	0.8
$d_{2}$	37.5 μm	32.1 μm
$d_{3}$	84.9 μm	497.53 μm
